# Anti-Inflammatory Action of an Antimicrobial Model Peptide That Suppresses the TRIF-Dependent Signaling Pathway via Inhibition of Toll-Like Receptor 4 Endocytosis in Lipopolysaccharide-Stimulated Macrophages

**DOI:** 10.1371/journal.pone.0126871

**Published:** 2015-05-27

**Authors:** Do-Wan Shim, Kang-Hyuck Heo, Young-Kyu Kim, Eun-Jeong Sim, Tae-Bong Kang, Jae-Wan Choi, Dae-Won Sim, Sun-Hee Cheong, Seung-Hong Lee, Jeong-Kyu Bang, Hyung-Sik Won, Kwang-Ho Lee

**Affiliations:** 1 Department of Biotechnology, College of Biomedical and Health Science, Konkuk University, Chungju, Chungbuk, Republic of Korea; 2 Division of Food Bioscience, College of Biomedical and Health Science, Konkuk University, Chungju, Chungbuk, Republic of Korea; 3 Division of Magnetic Resonance, Korea Basic Science Institute, Ochang, Chungbuk, Republic of Korea; Seoul National University College of Pharmacy, Republic of Korea

## Abstract

Antimicrobial peptides (AMPs), also called host defense peptides, particularly those with amphipathic helical structures, are emerging as target molecules for therapeutic development due to their immunomodulatory properties. Although the antimicrobial activity of AMPs is known to be exerted primarily by permeation of the bacterial membrane, the mechanism underlying its anti-inflammatory activity remains to be elucidated. We report potent anti-inflammatory activity of WALK11.3, an antimicrobial model peptide with an amphipathic helical conformation, in lipopolysaccharide (LPS)-stimulated RAW264.7 cells. This peptide inhibited the expression of inflammatory mediators, including nitric oxide, COX-2, IL-1β, IL-6, INF-β, and TNF-α. Although WALK11.3 did not exert a major effect on all downstream signaling in the MyD88-dependent pathway, toll-like receptor 4 (TLR4)- mediated pro-inflammatory signals were markedly attenuated in the TRIF-dependent pathway due to inhibition of the phosphorylation of STAT1 by attenuation of IRF3 phosphorylation. WALK11.3 specifically inhibited the endocytosis of TLR4, which is essential for triggering TRIF-mediated signaling in macrophage cells. Hence, we suggest that specific interference with TLR4 endocytosis could be one of the major modes of the anti-inflammatory action of AMPs. Our designed WALK11 peptides, which possess both antimicrobial and anti-inflammatory activities, may be promising molecules for the development of therapies for infectious inflammation.

## Introduction

Antimicrobial peptides (AMPs) are emerging as new anti-infective agents, with many natural and modified AMPs undergoing evaluation for therapeutic and commercial development. Some of these AMPs have reached the clinical trial stage [1–5]. The therapeutic potential of AMPs is not limited to their antimicrobial function, as they are an intrinsic component of innate immunity that has evolved in most living organisms. AMPs perform pleiotropic functions and target diverse host cellular processes that are frequently associated with human health and various diseases. The current working concept of AMPs defines them as multifunctional host defense peptides. Their therapeutic potential has expanded to include anti-inflammation, anti-tumor, insulinotropic, and myotropic activities [6,7].

**Fig 1:**
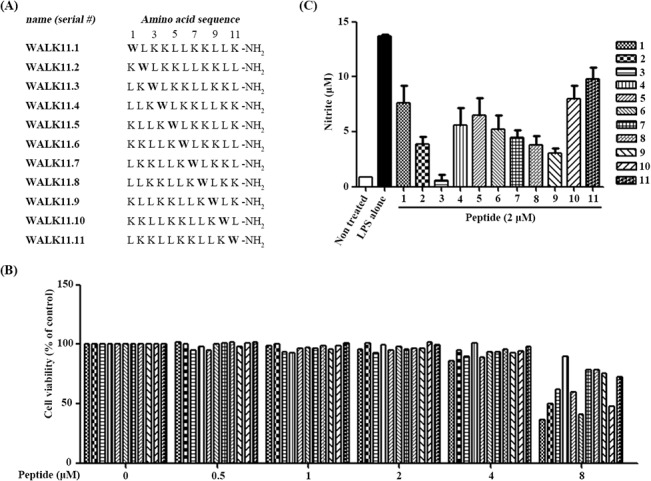
Initial screening of WALK11 peptide isomers. (A) The amino acid sequences of the WALK11 peptides tested in this study are presented, with their corresponding serial numbers (WALK11.1-WALK11.11), labeled according to the position of a single tryptophan (W; indicated in bold) in each sequence. (B) Cytotoxicity was evaluated by incubating RAW264.7 cells with each peptide (8–0.5 μM, two-fold serial dilutions) for 24 h, and the cell viability was assessed with an MTT assay as the percentage of surviving cells compared to that of the control cells. (C) The NO inhibitory effects of individual peptides were examined in cells pre-treated with each peptide (2 μM) for 1 h and stimulated by LPS (100 ng/ml) for 24 h. The culture supernatants were harvested and assayed for concentrations of nitrite, represented by bars with the mean ± SEM of independent experiments performed in triplicate.

Cationic, amphipathic α-helical peptides, which are attractive therapeutic targets for the treatment of infectious diseases, constitute a particularly abundant, widespread, and well-characterized class of naturally occurring AMPs [1–5]. Their membrane permeability leads to selective disruption of bacterial cell membranes, which is the most conserved and fundamental mode of action for their antimicrobial activity. The immunomodulatory properties of these AMPs are receiving a great deal of attention in studies aimed at developing new anti-inflammatory drugs [2]. In this context, we have recently developed a series of model peptide isomers, WALK11 peptides (tryptophan-containing, amphipathic-helical leucine/lysine undecapeptides) that share the same L_5_K_5_W formula and show potent antimicrobial activity [8,9] (Fig 1A). The aim of the present study was to investigate the anti-inflammatory potential of WALK11 peptides and to establish their mode of anti-inflammatory action.

Inflammation serves as a protective response to endo- and exogenous stimuli and is crucial for host survival; however, it is also responsible for many chronic inflammatory diseases [10, 11]. Activated macrophages, in particular, play a vital role in inflammation by producing various pro-inflammatory mediators [12,13]. Lipopolysaccharide (LPS), which belongs to the pathogen-associated molecular patterns (PAMPs), is the most widely used macrophage stimulator to induce rapid activation of innate immune responses in host cells [14]. Mechanistically, LPS is carried by the LPS-binding protein (LBP), followed by binding to the primary LPS receptor, CD14, which is expressed mainly on macrophages. An interaction between the LPS-LBP-CD14 complex and toll-like receptor-4 (TLR4), which is a major pathogen recognition receptor (PRR) of PAMPs, then triggers innate pro-inflammatory signaling pathways and influences adaptive immunity [15–17]. As LPS-induced signal pathways are generally considered anti-inflammatory targets, there has been an active search for potential anti-inflammatory agents that effectively inhibit pro-inflammatory signaling in macrophages. We tested the anti-inflammatory potential of the WALK11 peptides in an LPS-stimulated macrophage model and investigated the specific signal pathways responsible for the activity.

## Materials and Methods

### Materials and peptide preparation

ELISA kits (BD OptEIA TMSet) and TMB Substrate Reagent kits were purchased from BD Biosciences (Franklin Lakes, SD, USA). iNOS antibody was purchased from Upstate Biotechnology (CA, USA), and COX-2 and β-actin antibodies were purchased from Santa Cruz Biotechnology (CA, USA). Monoclonal antibodies against phospho-forms of p38, ERK, JNK, SEK1/MKK4, and STAT1 were purchased from Cell Signaling Technology Inc. (MA, USA). An ECL detection agent and polyvinylidene fluoride (PVDF) membrane were purchased from American Biosciences (NJ, USA). LPS and other chemicals were purchased from Sigma-Aldrich (St. Louis, MO, USA).

The WALK11 peptides were synthesized by Fmoc SPPS methods, as described previously [18]. Rink amide 4-methylbenzhydrylamine resin and 9-fluorenylmethoxycarbonyl (Fmoc) amino acids were obtained from Calbiochem-Novabiochem (La Jolla, CA). Other reagents used for peptide synthesis included trifluoroacetic acid (Sigma-Aldrich), piperidine (Merck), 1-O-benzotriazole-N,N,N’,N’-tetramethyl-uronium-hexafluoro-phosphate (Sigma-Aldrich), N-hydroxybenzotriazole hydrate (Sigma-Aldrich), N,N-diisopropylethylamine (Sigma-Aldrich) and dimethylformamide (peptide synthesis grade; Sigma-Aldrich). The final purity of the peptides (>95%) was assessed by reverse-phase HPLC on an analytical Vydac C_18_ column. The molecular masses of the purified peptides were determined using MALDI-TOF mass spectrometry at KBSI (Korea Basic Science Institute, Ochang, Korea). All the peptides were synthesized with C-terminal amidation to remove a negative charge at neutral pH, and the concentration of each peptide dissolved in its designated solvent was determined spectrophotometrically using the known value of molar absorptivity for tryptophan (5,500 M^-1^cm^-1^ at 280 nm), as these peptides commonly possess single tryptophan residues.

### Cell culture and viability test

The murine macrophage RAW264.7 cell line (ATCC, MD, USA) was maintained in DMEM media, supplemented with 10% heat-inactivated FBS and antibiotics (100 U/mL of penicillin and 100 μg/mL of streptomycin) at 37°C in humidified 5% CO_2_ and 95% air. An MTT assay was performed by culturing the cells in 96-well plates at a density of 1 × 10^4^ cells/well, followed by treatment with serially diluted WALK11 peptides and subsequent incubation for 24 h. The medium was then discarded, and 100 μL of DMEM medium containing MTT (500 μg/mL) were added to each well. After 4 h of incubation, the medium was discarded, and DMSO was added to each well to solubilize the formazan. The optical densities (550 nm) of the treated cells were compared to those of the control cells.

### Estimation of nitric oxide (NO) production

The nitrite concentration in the culture medium was determined by mixing a sample (100 μL) of culture medium with Griess reagent (100 μL, 1% sulfanilamide, 0.1% N-1-naphthyl ethylenediamine) and incubating for 10 min. The absorbance of the resulting chromophoric azo-derivative molecules was measured at 550 nm with a spectrophotometric microtiter plate reader (Molecular Devices Corp., Sunnyvale, CA, USA). Fresh culture medium was used as a blank in all the experiments. A range of dilutions of sodium nitrite was used for generation of a standard curve to determine the amount of nitrite in each sample.

### Enzyme-linked immunosorbent assay (ELISA)

The production of cytokine IL-6 and TNF-α was measured by an ELISA. The RAW264.7 cells in a 24-well plate (2 × 10^5^ cells per well) were pretreated with the peptide sample (2 μM) for 1 h and then stimulated with LPS (100 ng/mL) for 24 h, in the presence of serum. The supernatants were collected, and the cytokine concentrations were measured using a BD OptEIA TMSet, according to the manufacturer’s instructions. The generated color was measured at 450 nm using a microplate reader.

### Quantitative real-time PCR

The cells were cultured in the presence or absence of the peptide sample and LPS (100 ng/mL). After 4 h, total RNA was extracted by QIAzol lysis reagent (QIAGEN sciences, Maryland, USA). Then, 2 μg of total RNA was converted to cDNA using a First Strand cDNA Synthesis kit (Invitrogen, CA, USA). Real-time PCR, performed with 2X SYBR Green Supermix (Bio-Rad, Hercules, CA, USA), was analyzed using the iCycler program (Bio-Rad). The primers used were purchased from Bioneer (Seoul, Korea). For IFN- β, 5`-CAGCTCCAAGAAAGGACGAAC-3`and 5`-GGCAGTGTAACTCTTCTGCAT-3`were used. For HPRT, 5`-TCAGTCAACGGGGGACATAAA-3`and 5`-GGGGCTGTACTGCTT AACCAG-3`were used. The PCR condition was 7 min at 95°C, followed by 40 cycles of 95°C, 58–60°C, and 72°C for 30 s. The resulting mean -∆Ct values from each group were used to calculate the relative expression ratio of detected mRNA.

### Immunoblot analysis

The cells were lysed in 50 mM Tris-HCl buffer (pH 8.0) containing 1% Nonidet P-40 (NP-40), 150 mM sodium chloride, 0.5% sodium deoxycholate, 0.1% sodium dodecyl sulfate (SDS), and protease and phosphatase inhibitor cocktails. Equivalent amounts of cell lysates were separated by 10% SDS-PAGE and transferred to PVDF membranes, which were then blocked for 2 h at room temperature with PBS containing 5% fat-free dried milk. The membranes were incubated overnight with primary antibodies (diluted by 1/1000) at 4°C, followed by further incubation of the immune complexes with horseradish peroxidase-conjugated secondary antibodies for 1 h at room temperature. The membranes were developed using an ECL detection kit and visualized with a luminescent image analyzer (LAS-3000; Fujifilm, Tokyo, Japan).

### Endocytosis analysis

The endocytosis of TLR4 was estimated by plating the RAW264.7 cells in 6-well plates at a density of 5 × 10^5^ cells per well, followed by pretreatment with the peptide sample or a known endocytosis inhibitor, dynasore. After 1 h of incubation, the cells were stimulated with LPS and detached using a cell scraper. The cells were stained with PE-conjugated anti-TLR4/MD-2 antibody (MTS510; BD Pharmingen, San Diego, CA, USA) for 20 min at 4°C, according to the manufacturer’s recommendations. The endocytosis of FcεRI was estimated by pretreating RBL-2H3 cells (ATCC, MD, USA) in a 6-well plate (5 × 10^5^ cells per well) with mouse anti-DNP IgE (100 ng/mL, Sigma-Aldrich) at 37°C for 2 h. The cells were washed twice with PBS, re-suspended in opti-MEM, and pre-treated for 1 h with the peptide sample or dynasore. Internalization of FcεRI was induced by incubating the cells with DNP-human serum albumin (HSA) (100 ng/mL, Sigma) for 10 min at 37°C and stopped by subsequent incubation for 5 min at 4°C and washing with ice-cold PBS. The cells were then stained with FITC-conjugated anti-mouse IgE antibody (BD Pharmingen). The stained cells were washed with flow cytometry buffer (PBS containing 0.002 M EDTA, 0.1% sodium azide, and 2% FBS) and analyzed using a FacsCalibur flow cytometer (BD Pharmingen) with Cell Quest Pro software (BD Pharmingen). To assess mast cell degranulation, release of β-hexosaminidase in the RBL-2H3 cells was measured, as described previously [19].

### CD14 expression analysis

The RAW264.7 cells (5 × 10^5^ cells per well) were pre-incubated in the presence or absence of the peptide sample (2 μM) for 1 h at 37°C and washed with PBS, followed by staining with anti-CD14 (4C1; BD Pharmingen) antibody for 20 min at 4°C. The cells were washed with PBS and then stained with FITC-conjugated anti-Rat IgG antibody (sc-2011; Santa Cruz Biotechnology) for 20 min at 4°C. Finally, the cells were washed with flow cytometry buffer, and the cell surface expression of CD14 was estimated by flow cytometry.

### LPS binding analysis

The LPS-binding assay consisted of incubating the RAW264.7 cells with 1 μg/mL of FITC-conjugated LPS (*E*. *coli* 0111:B4; Sigma-Aldrich) in the presence or absence of the peptide sample or polymixin B (PMB) for 20 min. The cells were washed with PBS, and the binding of FITC-conjugated LPS to the cells was analyzed with a Spectramax M2e system (Molecular Devices, CA, USA). Fluorescence was measured at 538 nM, following excitation at 485 nm.

### Statistical analysis

All statistical analyses were calculated using GraphPad (San Diego, CA) Prism software.

## Results

### Initial screening of anti-inflammatory potential

**Fig 2:**
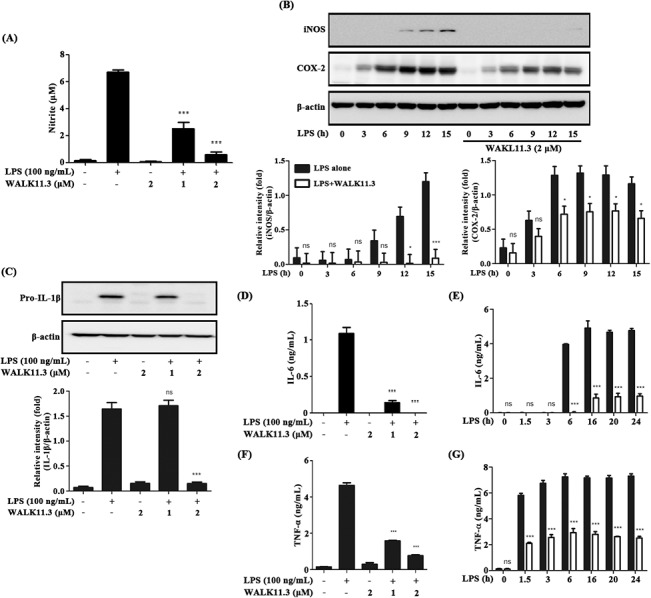
Effects of the WALK11.3 peptide on inflammatory mediators in LPS-stimulated RAW264.7 cells. The cells were pre-treated with WALK11.3 at different concentrations (0, 1 and 2 μM) for 1 h, before treatment with LPS (100 ng/mL) to induce inflammatory responses. (A) NO production was assayed after 24 h of incubation with LPS. iNOS and COX-2 protein levels (B) and pro-IL-1β expression (C) were analyzed by immunoblot analysis for 15 h during and 4 h after the LPS treatment. IL-6 (D and E) and TNF-α (F and G) production was measured with an ELISA, to monitor the dose-dependent (D and F; after 16 h and 4 h, respectively, of incubation with LPS) and time-dependent (E and G; with 2 μM peptide) effects of the pre-treated peptide. All bar graphs represent the mean ± SEM of three independent experiments. *p < 0.05 or ***p < 0.001 compared with the LPS- or WALK11.3-treated group (ns, non-significant). [Student’s *t*-test (A, C, D and E) or a one-way ANOVA with Bonferroni’s multiple comparison test (B)].

In our previous work, WALK11 peptide isomers (refer to the amino acid sequences in Fig 1A) exhibited quite variable hemolytic activities against human erythrocytes, although their antimicrobial potencies were similar [8]. Thus, prior to investigating the anti-inflammatory potential of the peptides, we performed an MTT assay to assess their cytotoxicity against RAW264.7 macrophage cells. The cytotoxic effects, which were detectable and variable depending on the individual peptides administered at concentrations of 8 μM or greater, were negligible for all peptides up to 4 μM (Fig 1B). Therefore, subsequent experiments were conducted at a non-toxic concentration of 2 μM. Analysis of the anti-inflammatory effects of the WALK11 peptides in the LPS-stimulated RAW264.7 cells showed that all the WALK11 peptide isomers exerted significant inhibitory effects on NO production but that their individual potency or efficacy varied (Fig 1C). Nearly complete, dose-dependent NO inhibition was observed in the cells treated with the WALK11.3 peptide (Fig 2A), whereas the treatment with WALK11.11 had the least inhibitory effect (Fig 1C). Therefore, WALK11.3, with the strongest anti-inflammatory potential, was selected for subsequent investigation of the anti-inflammatory action.

### Inhibition of pro-inflammatory mediator production

The effects of WALK11.3 on the production of representative pro-inflammatory mediators, including NO, COX-2, IL-1β, IL-6, and TNF-α, in the LPS-stimulated RAW264.7 cells were evaluated by an ELISA and immunoblotting. First, the inhibition of NO production (Fig 2A) was validated by confirming appreciable down-regulation of iNOS gene expression (Fig 2B) [20]. WALK11.3 also moderately attenuated the COX-2 protein level (Fig 2B) and markedly suppressed the LPS-induced production of pro-IL-1β (Fig 2C), IL-6 (Fig 2D and 2E), and TNF-α (Fig 2F and 2G). These results indicated that this peptide exerted anti-inflammatory activity by inhibiting LPS-induced production of inflammatory mediators in macrophage cells. Thus, we verified the anti-inflammatory action of WALK11.3 by examining its effects on pro-inflammatory signaling pathways.

### Partial suppression of MyD88-dependent pro-inflammatory signaling

**Fig 3:**
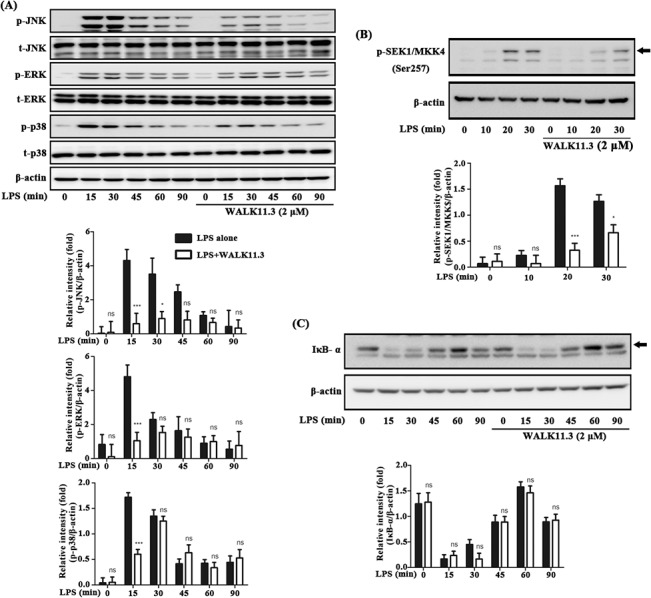
Effects of WALK11.3 on the MyD88-dependent signaling pathway in LPS-stimulated RAW264.7 cells. The cells were pre-treated with WALK11.3 (2 μM) for 1 h before the treatment with LPS (100 ng/mL). After the designated incubation time with LPS, the phosphorylation levels of three MAPKs were determined (JNK, ERK, and p38) (A), SEK1/MKK4 phosphorylation levels (B) and degradation of IκB-α (C) by immunoblot analysis. The bar graphs present the mean values ± SEM of three independent experiments. *p < 0.05 or ***p < 0.001 versus the peptide-treated cells (ns, non-significant). [One-way ANOVA with Bonferroni’s multiple comparison test (A-C)]

TLR4 signaling, which is primarily responsible for LPS activation of macrophages, consists of two different major downstream pathways: MyD88-dependent and MyD88-independent (TRIF-dependent) pathways [21]. The rapid onset of MyD88-dependent signaling, in particular, is accompanied by degradation of IκB-α and phosphorylation of mitogen-activated protein kinases (MAPKs), which results in the activation of the NF-κB and AP-1 transcription factors to promote the expression of pro-inflammatory mediators in macrophages [22]. We investigated the effects of WALK11.3 on the activation of the MyD88-dependent pathway by examining the phosphorylation of three MAPKs (JNK, ERK, and p38) in the LPS-stimulated RAW264.7 cells pre-treated with the peptide. The peptide treatment substantially inhibited the phosphorylation of JNK (Fig 3A) and inhibited SEK1/MKK4, the upstream signal of JNK (Fig 3B). Likewise, inhibition of the rapid onset (15 min in Fig 3A) of ERK phosphorylation was also appreciable and sustained. However, its inhibitory effect on p38 activation appeared to be relatively weak, as the significant inhibition at 15 min was not sustained to 30 min. In addition, at and after 30 min, the phosphorylation levels of ERK and p38 in the presence of the peptide were all comparable to those in the control cells where the peptide was not treated. Furthermore, the WALK11.3 treatment did not appear to have an inhibitory effect on NF-κB activation, as IκB-α was not degraded (Fig 3C). Collectively, these data indicate that the WALK11.3 peptide would be partly and/or incompletely able to suppress MyD88-dependent pro-inflammatory signaling in macrophages.

### Marked inhibition of TRIF-dependent signaling

**Fig 4:**
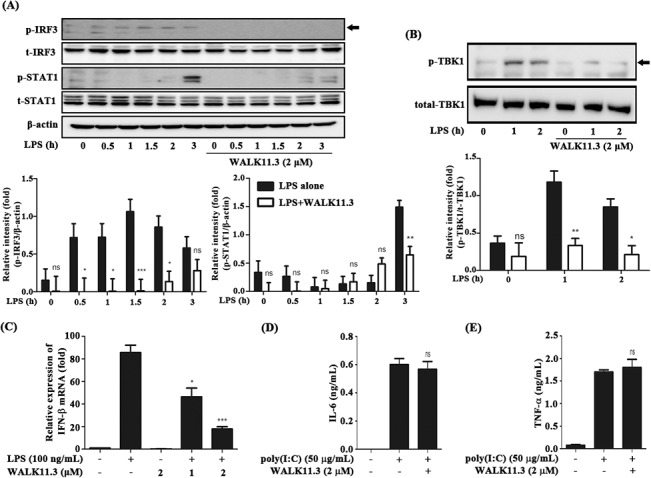
Effects of WALK11.3 on the MyD88-independent (TRIF-dependent) signaling pathway in LPS-stimulated RAW264.7 cells. The cells were pre-treated with WALK11.3 at the indicated concentrations for 1 h before the treatment with LPS (100 ng/mL) or poly(I:C) (10 μg/mL). After the designated incubation time with LPS, the phosphorylation levels of IRF3 (A), STAT 1 (A), and TBK1 (B) were estimated by immunoblot analysis. (C) The mRNA levels of IFN-β were determined by real-time PCR after 4h of incubation with LPS. After treatment with poly(I:C) for 24 h, the protein levels of IL-6 (D) and TNF-α (E) were measured by an ELISA. The bar graphs present the mean values ± SEM of three independent experiments. *p < 0.05, **p < 0.01 or ***p < 0.001 versus the peptide- or poly(I:C)-treated group (ns, non-significant). [Student’s *t*-test (C, D and E) or a one-way ANOVA with Bonferroni’s multiple comparison test (A and B)].

The TRIF-dependent signaling pathway mediated by TLR4 activates the transcription factor IRF-3 to induce IFN-β production, which, in turn, leads to the production of various pro-inflammatory mediators [23]. The secreted IFN-β is recognized by the Type I IFN receptor, triggering the activation of JAK/STAT1 signaling [24]. We investigated the inhibitory effect of WALK11.3 on the IRF-3 and STAT1 phosphorylation involved in TRIF-dependent signaling. The WALK11.3 peptide strongly inhibited the LPS-induced IRF-3 and STAT1 phosphorylation in the RAW264.7 cells (Fig 4A). Furthermore, the phosphorylation of TBK-1, which is located upstream of IRF3, was also significantly inhibited by the peptide treatment (Fig 4B). Quantitative real-time PCR results further corroborated the inhibition of STAT1 signaling, showing significant suppression of IFN-β transcription (Fig 4C). Collectively, the results suggested that the WALK11.3 peptide had potent anti-inflammatory activity, which was exerted by aggressive inhibition of TRIF-dependent signaling in the LPS-stimulated macrophages. The TRIF-dependent pathway is also essential for TLR3-mediated signaling, which, unlike TLR4, does not involve the Myd88-dependent pathway [25]. Macrophage activation induced by the TLR3-specific ligand, poly(I:C), was insensitive to the WALK11.3 peptide, as evidenced by unaltered levels of the pro-inflammatory cytokines IL-6 (Fig 4D) and TNF-α (Fig 4E). Thus, the WALK11.3 peptide showed specific inhibition of TLR4-mediated TRIF-dependent signaling.

### Inhibition of TLR4 endocytosis

**Fig 5:**
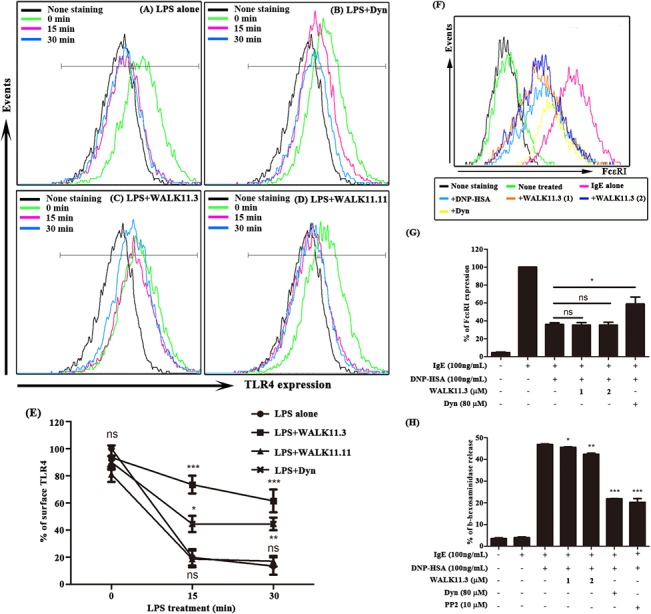
Effects of WALK11.3 on LPS-induced TLR4 endocytosis and DNP-HSA-induced FcεRI endocytosis. The RAW264.7 cells were incubated in the absence (A) or presence of dynasore (80 μM) (B), WALK11.3 (2 μM) (C), or WALK11.11 (2 μM) (D) for 1 h. After treatment with the LPS (100 ng/mL) for 30 min, flow cytometry was used to examine TLR4 endocytosis by measuring its surface expression on the cells. (E) The results are summarized as the mean fluorescence intensity of TLR4 staining at each time point. The error bars represent standard deviations from the average values of three independent experiments. (F) The endocytosis of FcεRI was also assessed by flow cytometry to detect the FcεRI level on the surface of the anti-DNP IgE-sensitized RBL-2H3 cells, which were stimulated with DNP-HSA (100 ng/mL) for 10 min, with or without pre-treatment (10 min) with WALK11.3 or dynasore. (G) The results are summarized as the mean fluorescence intensity of FcεRI/IgE staining. (H) DNP-HSA-induced degranulation in the RBL-2H3 cells was also estimated by measuring the activity of β-hexosaminidase in the cell culture media. The bar graphs present the mean values ± SEM of three independent experiments. *p < 0.05, **p < 0.01, or ***p < 0.001 compared with the DNP-HSA treated cells (ns, non-significant). [Student’s *t*-test (G and H) or a one-way ANOVA with Bonferroni’s multiple comparison test (E)].

Unlike TLR3, which is localized in endosomes, TLR4 is internalized from the plasma membrane into endosomes upon stimulation by LPS via a dynamin GTPase-dependent process [26]. We investigated whether the WALK11.3 peptide inhibited TLR4 endocytosis, which is essential for triggering TRIF-dependent signaling [27]. The LPS treatment rapidly reduced the expression of TLR4 on the RAW264.7 cell surface (Fig 5A), probably due to the internalization of TLR4 produced after LPS stimulation [17]. Dynasore, a known endocytosis blocker that inhibits dynamin GTPase [19], significantly inhibited this LPS-induced TLR4 endocytosis (Fig 5B). The WALK11.3 peptide also showed an appreciable inhibitory effect on TLR4 endocytosis, and it was even more effective than dynasore (Fig 5C). In contrast, the WALK11.11 peptide, which had the weakest anti-inflammatory potential of the 11 WALK11 peptide isomers tested (Fig 1C), had no significant effect on endocytosis (Fig 5D). The degree of inhibition of the different peptides is summarized in the histogram in Fig 5E.

We examined the effect of WALK11.3 on the internalization of IgE-sensitized FcεRI in mast cells to determine whether the inhibitory effect of WALK11.3 on endocytosis was specific to TLR4. As shown in Fig 5F and 5G, the internalization of FcεRI was efficiently induced in the IgE-sensitized RBL-2H3 cells by the DNP-HSA treatment. However, the WALK11.3 peptide had no significant effect, although the dynasore treatment significantly inhibited the DNP-HSA-induced FcεRI endocytosis. The WALK11.3 peptide also had no significant effect on DNP-HSA-induced mast cell degranulation, which is mediated by FcεRI internalization and is measurable by the release of β-hexosaminidase [19]; the treatment with dynasore or the src kinase inhibitor, PP2, suppressed DNP-HSA-induced mast cell degranulation (Fig 5H). In summary, the WALK11.3 peptide appeared to function by specifically inhibiting the TLR4 internalization process in macrophage cells.

### Effects on CD14 expression and LPS binding

**Fig 6:**
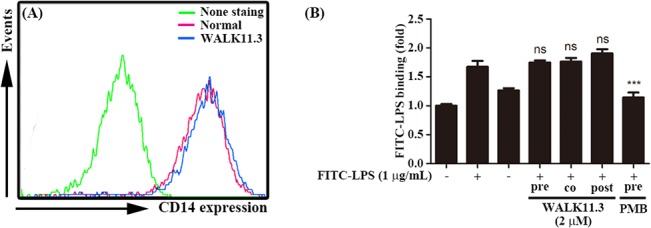
Effects of WALK11.3 on the CD14 expression level and LPS binding on RAW264.7 cells. The cells, pre-treated with or without WALK11.3 (2 μM) for 1 h at 37°C, were stained with anti-CD14 and subsequently with FITC-conjugated anti-Rat IgG antibodies. (A) The cell surface expression of CD14 was then evaluated by flow cytometry. (B) The LPS binding was also analyzed with a Spectramax M2e system to detect the FITC-conjugated LPS (20 min treatment) on the RAW264.7 cells, with or without pre-, co-, or 10 min post-treatment with WALK11.3 for 20 min. The effect of pretreatment with PMB was also compared in the same manner. *p < 0.05 versus the FITC-LPS treated group (ns, non-significant). [Student’s *t*-test (B)].

A recent report indicated that CD14 controls the LPS-induced endocytosis of TLR4 [17]. Therefore, we examined the effects of WALK11.3 on CD14 expression in the RAW264.7 cells. Flow cytometric analysis of CD14 indicated that the peptide treatment did not affect the expression of CD14 in the macrophage cells (Fig 6A). As CD14 functions as the primary receptor of LPS to activate TLR4 signaling [28,29], we examined whether WALK11.3 interfered with LPS binding to the macrophage cells. The known LPS binding inhibitor PMB significantly inhibited FITC-conjugated LPS binding to the cells. However, WALK11.3 did not inhibit FITC-conjugated LPS binding to the cells, regardless of the treatment order (pre-, co-, or post-treatment) (Fig 6B). Taken together, WALK11.3 did not directly block the binding of LPS to TLR4, but it inhibited the endocytosis of LPS-bound TLR4, leading to the blocking of TRIF-dependent signaling.

## Discussion

Many reports have confirmed the immunomodulatory potential of cationic, amphipathic helical AMPs, but the underlying molecular mechanism of their anti-inflammatory action remains controversial [28–31]. No generalized or conserved mode has been identified, whereas the mode of antimicrobial action is now relatively well established [1–5]. The great diversity of sequences of this peptide class, in particular, has prevented a thorough understanding of their immunomodulatory action. In the present study, we investigated the anti-inflammatory action of a *de novo* designed WALK11.3 peptide as a simple representative of cationic, amphipathic helical AMPs. The results verified the potent anti-inflammatory potential of this peptide, as shown by its ability to suppress the responses of macrophages upon the stimulation by LPS.

Many AMPs can bind to bacterial LPS and exert antimicrobial action through membrane permeation. Therefore, LPS scavenging or inhibition might be alternative explanations for the anti-inflammatory action of AMPs [30,31]. However, the present results for the WALK11.3 peptide indicate that the observed anti-inflammatory activity is not likely to be attributed to LPS scavenging, as LPS binding to macrophage cells was not significantly inhibited by the peptide, and not all the downstream pathways of LPS-stimulated TLR4 signaling were inhibited; for example, IκB-α degradation in the Myd88-dependent pathway was not inhibited by the peptide. Meanwhile, the observed LPS-neutralizing effects of WALK11.3 were attributable more significantly to the suppression of TRIF-dependent signaling, although the involvement of Myd88-dependent signaling could not be ruled out. The differences in the efficacy between iNOS suppression and COX-2 suppression (strong for iNOS and moderate for COX-2; Fig 2B) also support a different weighting between the two pathways, as iNOS induction requires IFN-β-induced STAT1 activation of the TRIF-dependent pathway, whereas NF-kB activation predominantly controls COX-2 expression in the Myd88-dependent pathway [32]. In addition, the initial attenuation of MAPK phosphorylation in the presence of the peptide (Fig 2A) is not sufficient to explain the long-standing inhibition of cytokine IL-6 and TNF-α production (Fig 3D–3G). In contrast, the present results indicated that WALK11.3 efficiently suppressed pro-inflammatory TRIF-dependent signaling by the specific inhibition of LPS-induced TLR4 endocytosis. It has been reported that the blocking of TLR4 endocytosis, which was achieved by CD14 knockout [17], resulted in the inhibition of JNK phosphorylation and reduced the production of cytokines, including IL-6 and TNF- α [33]. Thus, although JNK is one of the major components transducing Myd88-dependent signaling, the inhibition of JNK phosphorylation by WALK11.3 observed here could also be attributed to the inhibition of TRIF-dependent signaling. Likewise, TNF-α secretion, which is closely related to JNK phosphorylation [34], could be suppressed by deactivation of the TRIF-dependent pathway.

The manner in which the WALK11.3 peptide specifically inhibits TLR4 endocytosis remains to be established. CD14 is thought to play a central role in LPS-stimulated TLR4 endocytosis, and the transport of LPS to CD14 is mediated by the LBP [17,31]. Thus, one plausible mechanism for inhibition of TLR4 endocytosis by WALK11.3 might be strong competition with LPS for its binding sites on CD14 and/or LBP, as suggested for other antimicrobial peptides, such as LL-37 and cathelicidin peptides [28,29]. However, the present results indicated no significant effect of WALK11.3 on CD14 expression, and the peptide exerted only a very slight inhibitory effect on LPS binding to macrophage cells (Fig 6).

Taken together, the findings in this study indicate that the WALK11.3 peptide, a *de novo* designed cationic, amphipathic helical AMP, exhibited potent anti-inflammatory activity by efficiently deactivating the pro-inflammatory TRIF-dependent signaling pathway via inhibition of TLR4 endocytosis in LPS-stimulated macrophage cells. Thus, we propose that our WALK11 peptides, which show both antimicrobial and anti-inflammatory activities, are promising molecules for the development of therapies for infectious inflammation. In addition, as suggested previously [8,9], the properties of these rationally designed WALK11 peptides (*i*.*e*., their short length and simple amino acid composition) could be regarded as favorable for reducing manufacturing costs and facilitating pharmaceutical optimization for pharmaceutical and/or industrial production of bioactive peptide agents.
